# Preliminary profile of the gut microbiota from amerindians in the Brazilian amazon experiencing a process of transition to urbanization

**DOI:** 10.1007/s42770-024-01413-y

**Published:** 2024-06-24

**Authors:** Rodrigo M. Alencar, José G. Martínez, Valéria N. Machado, Juan F. Alzate, Cinthya P. Ortiz-Ojeda, Rosiane R. Matias, Denise C. Benzaquem, Maria C.F. Santos, Enedina N. Assunção, Evelyn C. Lira, Spartaco Astolfi-Filho, Tomas Hrbek, Izeni P. Farias, Cleiton Fantin

**Affiliations:** 1 Programa de Pós-graduação em Biotecnologia e Recursos Naturais da Amazônia, Universidade do Estado do Amazonas, Manaus, Brazil; 2 Grupo de investigación Biociencias, Facultad de Ciencias de la Salud, Institución Universitaria Colegio Mayor de Antioquia, Medellín, Colombia; 3 National Center for Genomic Sequencing, School of Medicine, Universidad de Antioquia, Medellín, Colombia; 4 Universidad Tecnológica del Perú, Lima, Peru; 5 Centro de Apoio Multidisciplinar, Universidade Federal do Amazonas, Manaus, Brazil; 6 Laboratório de Evolução e Genética Animal, Universidade Federal do Amazonas, Manaus, Brazil; 7 Department of Biology, Trinity University, San Antonio, USA

**Keywords:** Yanomami, 16s rRNA sequencing, Gut Microbiome, Manaus, westernization

## Abstract

The Yanomami are one of the oldest indigenous tribes in the Amazon and are direct descendants of the first people to colonize South America 12,000 years ago. They are located on the border between Venezuela and Brazil, with the Venezuelan side remaining uncontacted. While they maintain a hunter-gatherer society, they are currently experiencing contact with urbanized populations in Brazil. The human gut microbiota of traditional communities has become the subject of recent studies due to the Westernization of their diet and the introduction of antibiotics and other chemicals, which have affected microbial diversity in indigenous populations, thereby threatening their existence. In this study, we preliminarily characterized the diversity of the gut microbiota of the Yanomami, a hunter-gatherer society from the Amazon, experiencing contact with urbanized populations. Similarly, we compared their diversity with the population in Manaus, Amazonas. A metabarcoding approach of the 16 S rRNA gene was carried out on fecal samples. Differences were found between the two populations, particularly regarding the abundance of genera (e.g., *Prevotella* and *Bacteroides*) and the higher values of the phyla Bacteroidetes over Firmicutes, which were significant only in the Yanomami. Some bacteria were found exclusively in the Yanomami (*Treponema* and *Succinivibrio*). However, diversity was statistically equal between them. In conclusion, the composition of the Yanomami gut microbiota still maintains the profile characteristic of a community with a traditional lifestyle. However, our results suggest an underlying Westernization process of the Yanomami microbiota when compared with that of Manaus, which must be carefully monitored by authorities, as the loss of diversity can be a sign of growing danger to the health of the Yanomami.

**Supplementary Information:**

The online version contains supplementary material available at 10.1007/s42770-024-01413-y.

## Introduction

The Yanomami are one of the oldest indigenous tribes in the Amazon and are presumed to be direct descendants of the first people to colonize South America 12,000 years ago [1]. They are a hunter-gatherer society who live in isolated villages in the northern Amazon rainforest in Venezuela and Brazil but have recently experienced contact with Westernized societies in Brazil. The study of their microbiome became a matter of fundamental importance, as it showed the most diverse one ever seen in humans, clarifying the idea that a low bacterial diversity may be associated with several diseases in westernized populations [2], and reaffirming the need to study our human past through the study of microbiotas from isolated tribes [3, 4, 5].

Their diet is primarily based on seeds, roots, and fruits of the forest, fish, and occasionally meat [6]. The 258 Yanomami villages in Brazil are distributed across the territories of the states of Roraima and Amazonas, with some having access to nearby cities such as Barcelos (Amazonas) and Boa Vista (Roraima), while others are quite isolated [7]. The Yanomami Special Indigenous Sanitary District (DSEI-Y) is responsible for the health of the indigenous people and relies on the Indigenous Health Support House (CASAI) in Boa Vista to provide housing for indigenous people in need of medicines and more appropriate treatments.

The advancement of new-generation sequencing technology has made it possible to conduct studies on the human gut microbiota, leading to a greater understanding of how the composition of the gut microbiota is related to individual health [8, 9]. Some studies on human gut microbiota diversity have demonstrated the importance of maintaining high bacterial diversity in our microbiota, including a high ratio of Bacteroidetes to Firmicutes phyla, as observed in inhabitants of traditional communities such as hunter-gatherer societies in Africa, Peru, and Papua New Guinea [4]. Conversely, urbanized populations exhibit a lack of this diversity in their gut bacterial composition (including a low ratio of Bacteroidetes to Firmicutes phyla: dysbiosis), which can be an indicator of the health of these populations, as low bacterial diversity has been linked to obesity, diabetes, and various autoimmune diseases such as allergies, Crohn’s disease, and ulcerative colitis [10, 11].

Currently, there are several studies related to identifying the composition of the gut microbiota of traditional and remote communities. These studies allow us to discover new evidence about the ancestral states of microbiotas and the historical changes in host-microbe interactions [12–18], providing a better understanding of how our gut microbiota has changed over time. However, few studies are investigating Amerindian communities [1, 4, 10, 19], particularly those from the Brazilian Amazon such as the Yanomami; which have had troubles such as “*bias imposed by an underrepresented sampling of Yanomami*” [20], which includes a punctual sampling of single villages but lacks representation across several ones experiencing westernization of their lifestyle through dietary changes and the introduction of antibiotics and other chemicals, affecting their microbial diversity and threatening their existence.

In this study, we tested the hypothesis that, although previous studies have shown isolated populations of Yanomami in Venezuela to have the greatest gut microbiota diversity known to date [1], the westernization of the Yanomami in northern Brazil may be decreasing their diversity to the point of resembling that of the largest urban center in the Amazon, the city of Manaus. To investigate this, we taxonomically characterized the gut microbiota of a traditional Amerindian society within the Brazilian Amazon and compared it with the gut microbiota of an urbanized population in Manaus, Amazonas. We used sequencing of the 16 S rRNA partial gene (V1-V2 region) from fecal samples collected from Yanomami individuals representing nine villages and from individuals in Manaus.

## Materials and methods

### Ethical aspects and sample collection

A partnership was initially established with the Yanomami Special Indigenous Sanitary District (DSEI-Y) in the city of Boa Vista, Roraima, to seek their assistance, follow-up, and surveillance of the research process, without the need to enter indigenous lands or have direct contact with the Yanomami. The participation of the Yanomami people was authorized by the coordinator of DSEI-Y and by the indigenous leader, Mr. Davi Kopenawa. The protocol of this study was approved by the Research Ethics Committee of the Universidade do Estado do Amazonas (No. 3.749.298). With the assistance of the medical team from the Indigenous Health Support House (CASAI) in Boa Vista and a bilingual interpreter, the objectives and importance of the study, as well as the sample collection procedures, were explained. Consent from the participating indigenous individuals was obtained using a fingerprint.

**Fig. 1:**
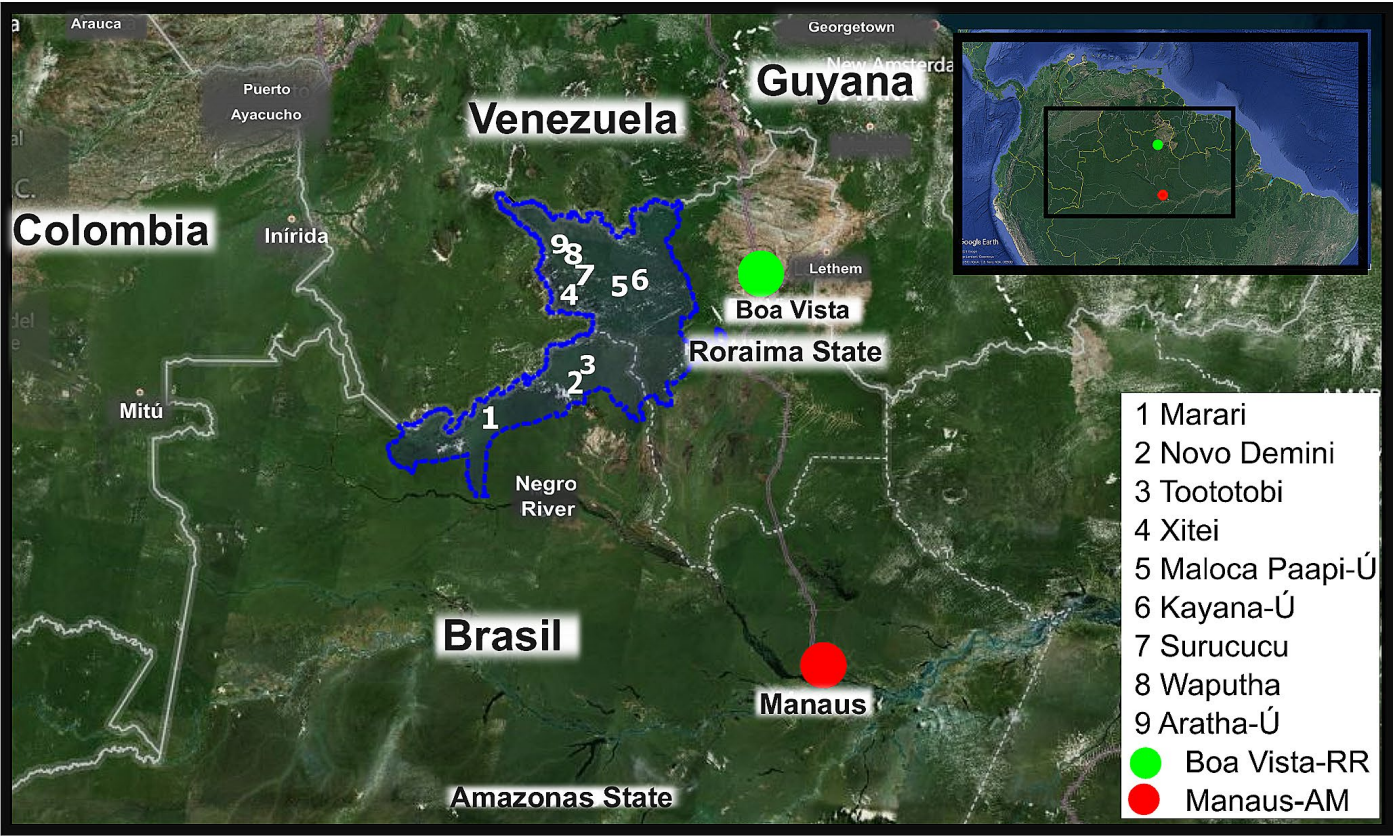
Map of the Yanomami territory and the localization of Manaus-Amazonas (AM) and Boa Vista-Roraima (RR). The Yanomami territory is demarked in the blue line in Brazil and Venezuela. The numbers represent the villages where the individuals in this study live. The green point is the CASAI localization, in Boa Vista-RR. The red point is the Manaus-AM localization Sources: CASAI and Google Earth

The experimental groups consisted of: (1) Individuals from Yanomami villages distributed across the territories of Roraima and Amazonas, undergoing a transition to urbanization; and, (2) Inhabitants of the city of Manaus, Amazonas (Fig. 1; Online Resource 2). The collection of fecal samples from indigenous individuals was conducted at CASAI in Boa Vista, where 18 healthy Yanomami individuals were selected: two infants aged up to 12 months; six children up to 10 years old; five young adults up to 35 years old; and five adults aged 50 to 65 years, categorized here as seniors. Stool samples were collected by the indigenous individuals themselves with the assistance of the CASAI medical team. From the Manaus population group, 12 samples were collected from individuals in the same age groups as the indigenous people: one infant, four children, four young adults, and three seniors (see Online Resource 2). Following collection, the samples were immediately frozen at -20 °C and remained frozen until the DNA extraction stage. After that, the samples were returned to their owners, as agreed.

### DNA extraction and next-generation sequencing

Bacterial DNA was isolated using the PowerSoil DNA Isolation Kit (MoBio) [1] following the manufacturer’s instructions. For amplification, we conducted Polymerase Chain Reaction (PCR) targeting the variable region V1-V2 of the 16S rRNA gene [21], using the bacterial primers 27F (V1 forward primer; 5’-AGAGTTTGATCCTGGCTCAG-3’) and 338R (V2 reverse primer; 5’- TGCTGCCTCCCGTAGGAGT-3’). The PCR reaction included 1 U of Taq DNA polymerase (Thermo Fisher Scientific), Taq DNA polymerase 1X buffer (Thermo Fisher Scientific), 1.5 mM MgCl2 (Invitrogen), 5 pmol of the forward primer containing the “A” adapters, 5 pmol of the reverse primer containing the “P1” adapters, 0.2 mM dNTPs (Invitrogen), and 25 ng of DNA, for a final volume per reaction of 30 µL. The samples were subjected to an automatic thermal cycler with the following profile: initial denaturation at 95 °C for 30 s; 30 cycles of denaturation at 94 °C for 15 s, annealing of primers at 64 °C for 15 s, and extension at 72 °C for 15 s; followed by a final extension at 72 ºC for five minutes. Four PCR replicates were performed for each individual, resulting in approximately 120 µL of mixed PCRs in the final amplicon library. Subsequently, each mixed PCR per individual was first purified using the magnetic beads method, followed by purification using the column method (Illustra GFX PCR DNA and Gel Band Purification kit, USA). Once purified, each library was quantified using fluorimetry (Qubit® 2.0 Fluorometer, Life Science, USA) following the manufacturer’s instructions. The libraries were then merged in an equimolar manner (100 ng) and subjected to clonal amplification using Ion One Touch following the manufacturer’s instructions. Sequencing was performed on an IonTorrent PGM (Life Technologies, USA) using the 400 bp kit on the 318 IonPGM chip.

### Computational biology and statistical analysis

The raw reads were converted to the FASTQ format and filtered (sequences with Phred Score > 25) using PRINSEQ [22]. Data analysis was conducted using Mothur [23]. Subsequently, individuals were demultiplexed by age group using the approach of “pooled samples”, as developed by Ray et al. (2019) [24]. This allowed us to analyze the gut microbiota based on biological age pools: Babies, Children, Adults, and Seniors. The sequences were aligned with the SILVA reference database [25], followed by filtering to remove duplicates. Sequences were then classified and grouped into taxa or Operational Taxonomic Units (OTUs), considering a similarity of at least 97% [26] to be grouped into the same OTU at the genus level. Relative abundance was calculated based on the representative contigs of each OTU. Details of filter parameters in Mothur can be found in Online Resource 1.

The barplot and ggplot2 R packages were used in R-studio v3.6.1 for statistical analyses and general data plotting, including descriptive statistics (mean and standard deviation) and frequency bars. The *.shared file (containing age group and relative abundance of each OTU) was used as input for Principal Component Analysis (PCA) and microbiota similarity analysis (based on Cluster Analysis of Observations) in Minitab v.18, using a complete linkage method with the Euclidean distance matrix. IBM SPSS Statistics v.22 was utilized to configure a t-test of independent samples (T-student) after analyzing normality with the Shapiro-Wilk test to detect differences in Ace diversity indices between Yanomami and Manaus age groups.

Differences in the relative abundance of Firmicutes and Bacteroidetes within and between Yanomami and Manaus populations were explored using a non-parametric multiple comparison test of Kruskal-Wallis. Additionally, the Venn command in Mothur was employed to generate a Venn diagram from data provided in the *.shared file, enabling comparison of shared richness among groups. All comparisons were conducted at a significance level of 0.05 and a confidence level of 95% for the aforementioned comparison tests. The normalized absolute abundance for all identified taxa was calculated based on the frequency of sequences found, divided by the number of individuals in each age group, allowing calculation of relative abundance for each group.

## Results

**Fig. 2:**
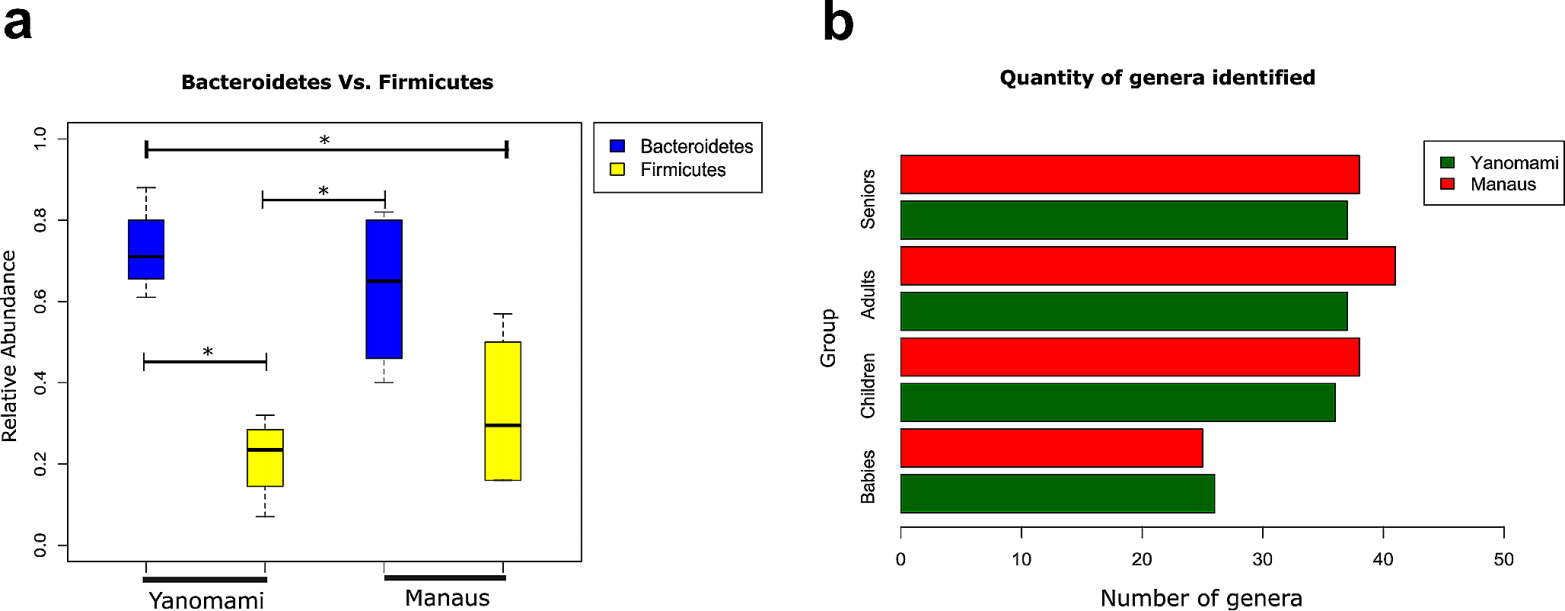
(a) Boxplot of the comparison of abundance between Bacteroidetes and Firmicutes found in the gut microbiota of the Yanomami and Manaus populations. Asterisks are representing statistical differences at 5% of significance for the Kruskal-Wallis test. (b) Comparison of the number of bacterial genera identified between the Yanomami and Manaus populations

After next-generation sequencing, a total of 6.2 million raw reads were obtained. Following filtering to remove adapter sequences, contamination, and low-quality reads using PRINSEQ and Mothur programs, a total of 2,863,034 clean reads were obtained, with an average length of 384 bp (ranging from 250 to 550 bp). Regarding phyla identification, both the Yanomami and Manaus populations showed a greater abundance of Bacteroidetes over Firmicutes [Yanomami variation between age groups: 61–88% (Bacteroidetes) and 7–32% (Firmicutes); Manaus variation between age groups: 40–82% (Bacteroidetes) and 16–57% (Firmicutes)]. The median Firmicutes/Bacteroidetes (FB) ratio was 0.24 and 0.55 for the Yanomami and Manaus populations, respectively. However, only in the Yanomami population was Bacteroidetes significantly dominant over Firmicutes (*p* = 0.02092) (Fig. 2a).

The identification of microbiota from Yanomami and Manaus age groups reached the genus level with 147 OTUs, 54 of which were nominally identified (see Online Resource 3). Among these genera, 40 were shared between the two populations, while three were exclusive to the Yanomami and 11 to Manaus. In Yanomami, the genera *Peptostreptococcus, Succinivibrio*, and *Treponema* were found, whereas in individuals from Manaus, the genera *Clostridium XlVa*, *Clostridium XVIII*, *Mitsuokella, Flavonifractor, Catenibacterium, Anaerostipes, Pseudoflavonifractor, Victivallis, Parasutterella, Cloacibacillus*, and *Megamonas* were identified. The number of genera found between the two populations was very similar, particularly in the Babies and Seniors groups considering both populations (Fig. 2b).

**Fig. 3:**
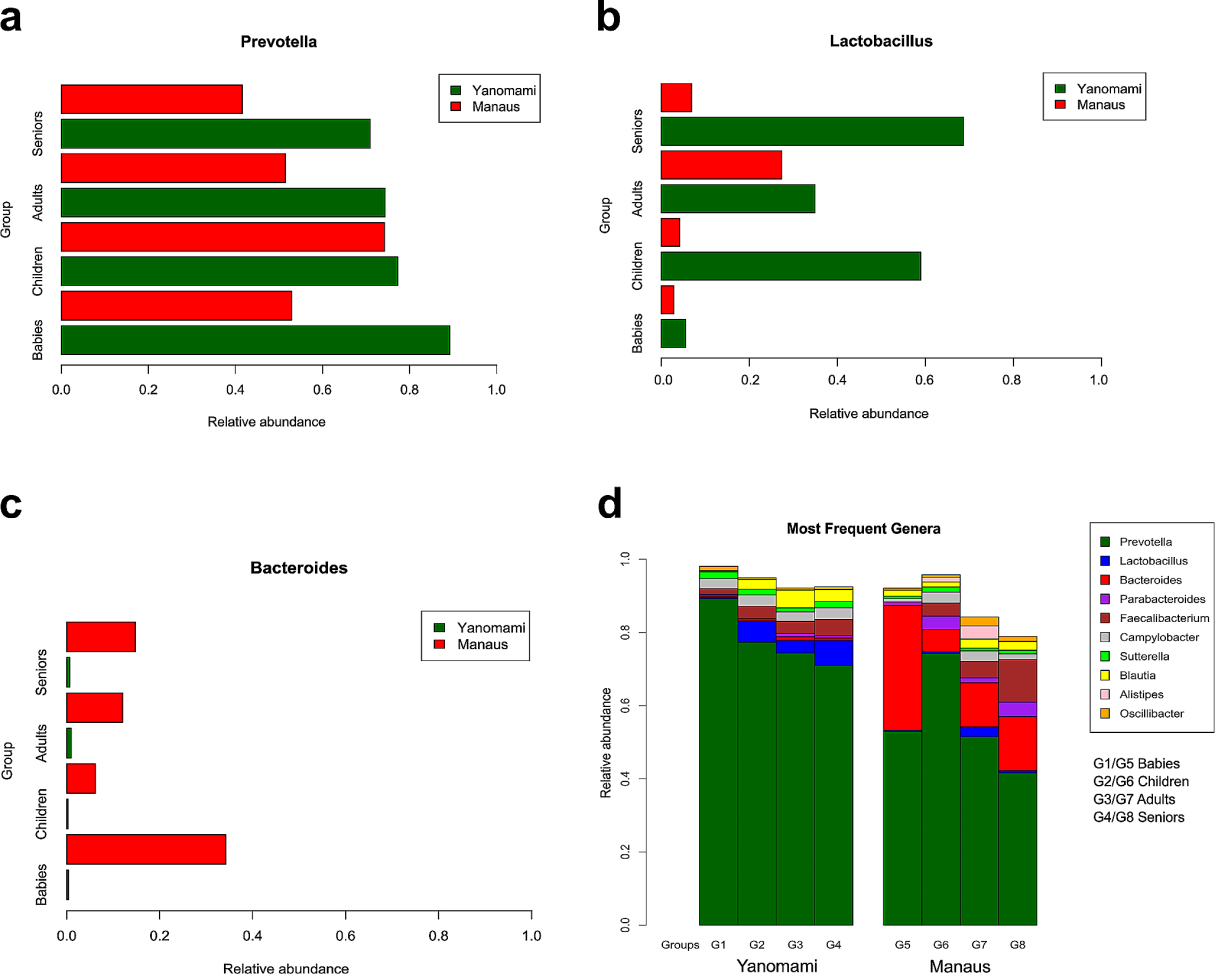
The graph shows the relative abundance of OTUs found in the gut microbiota of the Yanomami and Manaus population by age group for (a) *Prevotella*, (b) *Lactobacillus*, (c) *Bacteroides*, and (d) Ten main genus in the two populations

Regarding the composition of the Yanomami gut microbiota, the largest OTU belonged to the *Prevotella* genus, which exhibited greater abundance in the Yanomami microbiota compared to Manaus. However, when analyzed by age group, it was found that only children from Manaus had an abundance relatively close to that of the Yanomami population (Fig. 3a). The second most abundant genus found in the Yanomami gut microbiota was *Lactobacillus* (Fig. 3b), which was present in higher abundance in Yanomami individuals than in those from Manaus. Conversely, the population of Manaus exhibited a high abundance of the genus *Bacteroides*, while the Yanomami population showed very low levels across all age groups (Fig. 3c). Differences in the relative abundance of the 10 most abundant genera between populations were observed, with the genus *Bacteroides* serving as a marker for Manaus (Fig. 3d).

**Fig. 4:**
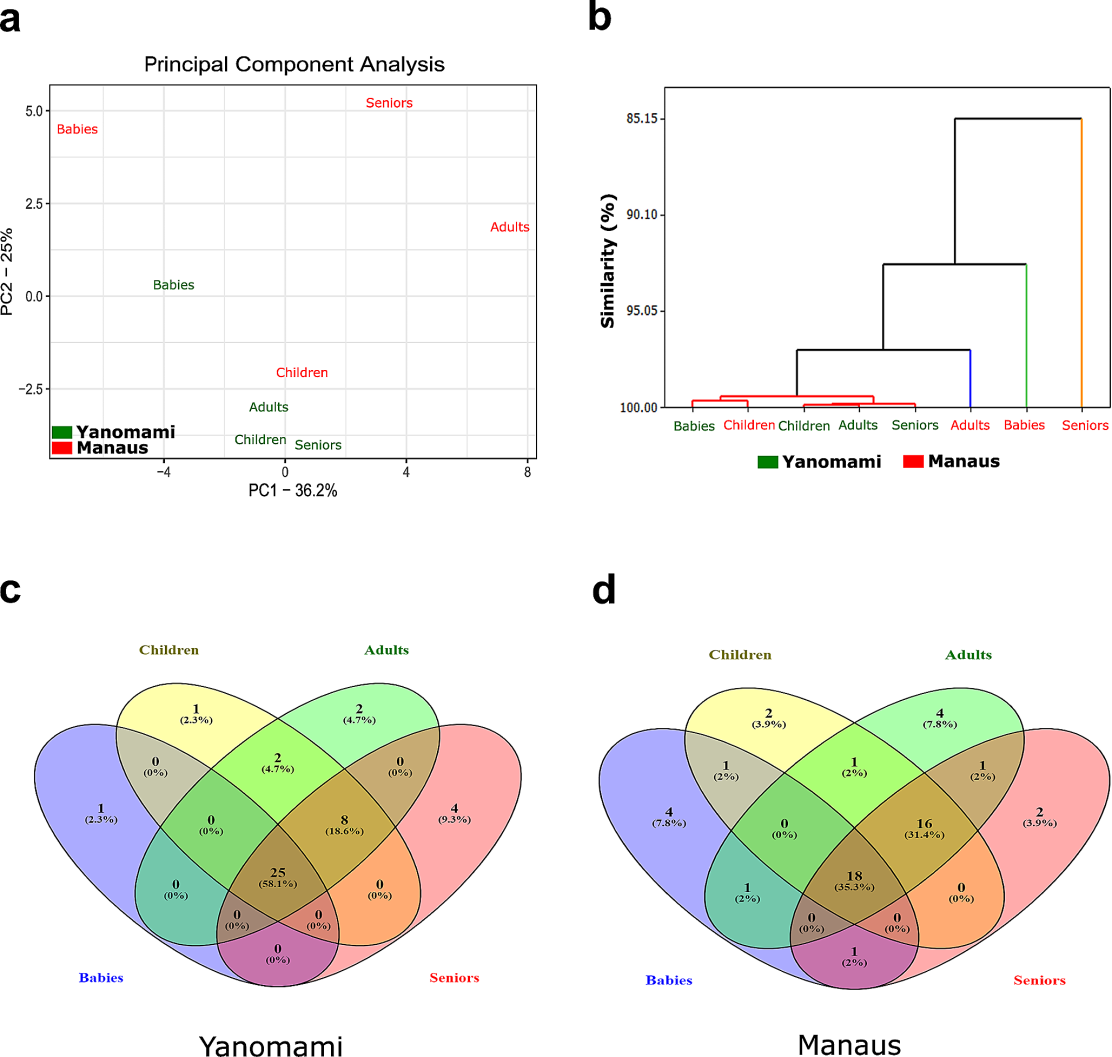
(a) Principal component analysis (PCA) between the groups by age group analyzing the abundance of the genera identified in the Yanomami and Manaus individuals. (b) Dendrogram of similarity of the gut microbiota among the age groups for Yanomami and Manaus in terms of OTUs abundance. (c) Venn diagram among the OTUs found in the age groups in Yanomami microbiota. (d) Venn diagram among the OTUs found in the age groups in Manaus microbiota

Principal Component Analysis (PCA) did not completely or significantly separate the Yanomami and Manaus groups based on these microbial composition variables (Fig. 4a). The total variance in the PCA was primarily explained by two components (PC1 = 36.2% and PC2 = 25%). However, the similarity analysis approach effectively discriminated the Yanomami population from that of Manaus (Fig. 4b). Indeed, the Yanomami microbiota exhibited greater similarity among its members compared to the Manaus microbiota, sharing the most genera among all age groups (58.1%) (Fig. 4c), as opposed to Manaus (35.3%) (Fig. 4d). On the other hand, alpha diversity levels indicated that the Yanomami population had bacterial diversity between 1.2 and 2 times greater than that of individuals from Manaus across all age groups. However, no statistical difference was found between them (*p* = 0.3972, t = 0.9115).

## Discussion

The Yanomami living in Brazilian territory represent an essential ethnic group contributing to the cultural and biological diversity of Amazonian indigenous communities. These people, with traditional lifestyles, offer a unique opportunity to gain insights into the composition of gut microbiota in an ancestral civilization (dating back approximately 12,000 years) undergoing transition to urbanization. The results of this study indicate that the Yanomami share certain bacteria with isolated Yanomami populations in Venezuela [1] and other Yanomami from Amazonas [20], including *Prevotella, Treponema, Lactobacillus, Campylobacter, Alistipes*, among others.

Despite the ongoing transition, the composition of the microbiota in the Yanomami population still exhibits significant differences compared to the population of Manaus, consistent with findings from various studies comparing industrialized populations with traditional communities experiencing urbanization and cultural changes [4, 10, 13]. However, when assessed in terms of bacterial diversity, the Yanomami and individuals from Manaus demonstrated similar levels of alpha diversity, contrary to findings in previous studies. This could be attributed to the adoption of some Western habits within indigenous villages, such as traveling to cities to purchase rice, poultry for breeding, alcoholic beverages, and household goods. Additionally, access to medical care and treatment with pharmaceuticals may influence changes in gut microbiota diversity. This sporadic yet progressive contact between the Yanomami and urbanized populations may lead to homogenization of their microbiotas, as decreasing diversity is often associated with the industrialization process [16, 27].

The gut microbiota of the Yanomami exhibited a significantly greater abundance of the phylum Bacteroidetes over Firmicutes, with a Firmicutes/Bacteroidetes (FB) ratio of 0.24. This ratio, as described by Magne et al. (2020), is consistent with populations adhering to more traditional lifestyles, such as those in Indian and Pakistani villages, in contrast to Westernized populations like those in the United States or the United Kingdom, where the ratio is consistently greater than 1 (see Online Resource 4 for further details). A similar pattern was recently observed in Colombian Agropastoralist indigenous populations in Siminke, Northern Colombia, with ratios of 0.66, indicating a dominant presence of Bacteroidetes [28]. Comparable results were also found in the Hadza hunter-gatherer population in Africa, characterized by a relatively higher abundance of Bacteroidetes and lower abundance of Firmicutes [12], as well as in the Tunapuco population, a traditional agricultural community from the Andean highlands in Peru [4].

However, this compositional profile is not a universal rule among traditional populations worldwide. From Africans to Amerindians, populations are predominantly enriched by Firmicutes [4, 12, 20], with some exceptions, such as the Yanomami population. For example, Firmicutes have been found to dominate the microbiota of the Matses, a remote hunter-gatherer population from the Peruvian Amazon [4]. Indeed, Firmicutes are directly associated with a plant-based diet [29, 30], and therefore, these variable profiles among traditional populations may also be directly linked to dietary factors [31].

Although Manaus is an urban center, its FB ratio was 0.55, twice that of the Yanomami, but still within the range of a population with a non-westernized lifestyle [< 1 (see Online Resource 4)]. This pattern mirrors that found in urban populations of Colombia (specifically in the city of Valledupar) when compared to nearby Agropastoralist indigenous populations in Siminke (FB = 0.66 for both). Despite the urban populations of Manaus and Valledupar not being direct descendants of indigenous people and having socially differentiated lifestyles, including the consumption of industrialized foods, much of their eating behavior, particularly diet, and culture is still shared. This shared cultural heritage could be influencing the maintenance of a microbiota profile similar to that of traditional populations in urban populations, especially in developing countries. Indeed, despite geographic and apparent lifestyle differences between the Yanomami and Manaus populations, levels of diversity showed that the composition of their gut microbiota was very similar.

However, PCA analyses revealed a more homogeneous microbiota among the Yanomami, indicating a stronger cultural adherence to sourcing food through hunting and ancestral customs across age groups compared to the Manaus population.

On the other hand, the predominant bacterial genus identified in the Yanomami gut microbiota was *Prevotella*. *Prevotella* is commonly found in abundance in the gut microbiota of traditional populations with diets rich in plant-derived carbohydrates, particularly fibers [1, 4, 12, 32]. Such populations are known to exhibit higher bacterial diversity in their microbiomes [33], which favors the colonization of *Prevotella* [12], contrasting with the composition found in industrialized populations where *Bacteroides* genus is typically dominant [1, 32, 34]. In our study, we observed a greater abundance of *Bacteroides* in Manaus, which is often associated with an animal-based diet [29, 30].

This pattern is consistent with the initial study of uncontacted Yanomami from Venezuela, where their fecal microbiota was characterized by high levels of *Prevotella* and low levels of *Bacteroides*, similar to Guahibo Amerindians, Malawians, and African hunter-gatherers [1, 35]. However, it contrasts with observations in subjects from the United States in the same study [1], and with the initial study of Yanomami from Brazil [20], where the *Prevotella* genus was not significantly associated with this group. The latter study suggested that these differences could be attributed to the bias resulting from underrepresented sampling of the Yanomami, such as punctual sampling (from one village) with a sample size of 15, and factors such as spatial and temporal variations (e.g., seasonality) that notably impact hunter-gatherers. In our study, we sampled at least nine villages, covering a significant portion of the Yanomami distribution area along the northern border of Brazil.

Despite these differences, a greater abundance of *Prevotella* was also observed in Manaus, underscoring how the two populations in our study may still share similarities in their microbiota, possibly due to current contact or maintenance of similar food sources and cultural practices. The Yanomami had their first contact with non-indigenous populations in the 1960s when missionaries began working in their territory. In subsequent years, the discovery of gold led to mining incursions into their territory, resulting in increasingly frequent contact with westernized populations up to the present day [7, 36].

The second genus found with greater abundance in the Yanomami population was *Lactobacillus*. These beneficial bacteria are abundant in the microbiota of traditional communities like the Yanomami [1, 20], and hold significant potential to provide health benefits to such communities, particularly the Yanomami, as they can be used for the treatment and prevention of diarrhea [37]. Therefore, they serve as a natural protective element in their gut microbiota by potentially inhibiting the proliferation of harmful bacteria [38].

Despite contact with urbanized populations, both the Yanomami and Manaus populations exhibited some exclusive bacteria in their gut microbiota. For the Yanomami, two of these exclusive genera (*Succinivibrio* and *Treponema*) are common among traditional populations worldwide, regardless of whether they are Amerindian or not. These genera were found in the gut microbiota of Yanomami adults and seniors. *Succinivibrio* is typically present exclusively in the gut microbiota of hunter-gatherer communities, as it has been identified in traditional populations in Africa [12, 39], as well as in two Amerindian populations in Peru [4]. Similarly, bacteria of the genus *Treponema*, found in children from traditional communities in Burkina Faso, hunter-gatherer communities in Tanzania, and Amerindian populations in Peru and Venezuela, serve as markers of traditional communities and are absent in urbanized populations in the United States and European countries [1, 4, 12, 14, 32, 39].

In conclusion, the composition of the Yanomami gut microbiota still retains bacteria characteristic of a community with a traditional lifestyle. Thus, the gut microbiota of the Yanomami is distinguished from that of individuals in Manaus by taxonomic differences, either due to exclusive or differentially abundant OTUs. However, alpha-diversity indices, when compared with Manaus, suggest a westernization process among the Yanomami group, which warrants careful monitoring by authorities, as the loss of diversity could signal growing health risks for the Yanomami.

## Electronic supplementary material

Below is the link to the electronic supplementary material.

Supplementary Material 1

Supplementary Material 2

Supplementary Material 3

Supplementary Material 4

## Data Availability

The 16 S rRNA metagenomic raw reads dataset was deposited into the GenBank (NCBI) under the Sequence Read Archive (SRA) BioProject PRJNA812515 (BioSamples accessions SAMN26417896 - SAMN26417903).
