# Supplementary material for: Preliminary profile of the gut microbiota from amerindians in the Brazilian amazon experiencing a process of transition to urbanization
Source: Braz J Microbiol. 2024 Jun 24;55(3):2345–54. doi: 10.1007/s42770-024-01413-y (PMC11405645; doi:10.1007/s42770-024-01413-y)
Supplement: Supplementary file 1 — Supplementary Material 1 [file 42770_2024_1413_MOESM1_ESM.docx]

**Preliminary Profile of the Gut Microbiota from Amerindians in The Brazilian Amazon Experiencing a Process of Transition to Urbanization**

Brazilian Journal of Microbiology

Rodrigo M. Alencar^1^, José G. Martínez^1,2*^, Valéria N. Machado^1^, Juan F. Alzate^3^, Cinthya P. Ortiz-Ojeda^1,4^, Rosiane R. Matias^1^, Denise C. Benzaquem^1^, Maria C.F. Santos^1^, Enedina N. Assunção^5^, Evelyn C. Lira^5^, Spartaco Astolfi-Filho^5^, Tomas Hrbek^6^, Izeni P. Farias^6^, Cleiton Fantin^1^

^1^ Programa de Pós-graduação em Biotecnologia e Recursos Naturais da Amazônia, Universidade do Estado do Amazonas, Manaus, Brazil; ^2^ Grupo de investigación Biociencias, Facultad de Ciencias de la Salud, Institución Universitaria Colegio Mayor de Antioquia, Medellín, Colombia; ^3^ National Center for Genomic Sequencing, School of Medicine, Universidad de Antioquia, Medellín, Colombia; ^4^ Universidad Tecnológica del Perú, Lima, Peru; ^5^ Centro de Apoio Multidisciplinar, Universidade Federal do Amazonas, Manaus, Brazil; ^6^ Laboratório de Evolução e Genética Animal, Universidade Federal do Amazonas, Manaus, Brazil.

***** Corresponding author e-mail: [jose.martinez@colmayor.edu.co](mailto:jose.martinez@colmayor.edu.co)

**Online Resource 1.** Supplementary text about the pipeline/script used for the 16s raw reads processing and bacterial microbiota identification using the Mothur software.

seqtk seq -r in.fq > out.fq

mothur > make.contigs(file=yanomami16s2.files, processors=50)

mothur > summary.seqs(fasta=yanomami16s2.trim.contigs.fasta)

mothur > screen.seqs(fasta=yanomami16s2.trim.contigs.fasta, group=yanomami16s2.contigs.groups, maxambig=0, maxlength=433)

mothur > summary.seqs()

mothur > unique.seqs(fasta=yanomami16s2.trim.contigs.good.fasta)

mothur > count.seqs(name=yanomami16s2.trim.contigs.good.names, group=yanomami16s2.contigs.good.groups)

mothur > summary.seqs(count=yanomami16s2.trim.contigs.good.count_table)

mothur > pcr.seqs(fasta=silva.bacteria/silva.bacteria.fasta, start=1044, end=8188, keepdots=F, processors=40)

mothur > align.seqs(fasta=yanomami16s2.trim.contigs.good.unique.fasta, reference=silva.bacteria/silva.bacteria.pcr.fasta)

mothur > summary.seqs(fasta=yanomami16s2.trim.contigs.good.unique.align, count=yanomami16s2.trim.contigs.good.count_table)

mothur > screen.seqs(fasta=yanomami16s2.trim.contigs.good.unique.align, count=yanomami16s2.trim.contigs.good.count_table, summary=yanomami16s2.trim.contigs.good.unique.summary, start= 2, end=5418, maxhomop=6)

mothur > summary.seqs()

mothur > filter.seqs(fasta=yanomami16s2.trim.contigs.good.unique.good.align, vertical=T, trump=.)

mothur > unique.seqs(fasta=yanomami16s2.trim.contigs.good.unique.good.filter.fasta, count=yanomami16s2.trim.contigs.good.count_table)

mothur > pre.cluster(fasta=yanomami16s2.trim.contigs.good.unique.good.filter.unique.fasta, count=yanomami16s2.trim.contigs.good.unique.good.filter.count_table, diffs=3)

mothur > chimera.vsearch(fasta=yanomami16s2.trim.contigs.good.unique.good.filter.unique.precluster.fasta, count=yanomami16s2.trim.contigs.good.unique.good.filter.unique.precluster.count_table, dereplicate=t)

mothur > remove.seqs(fasta=yanomami16s2.trim.contigs.good.unique.good.filter.unique.precluster.fasta, accnos=yanomami16s2.trim.contigs.good.unique.good.filter.unique.precluster.denovo.vsearch.accnos)

mothur > summary.seqs(fasta=yanomami16s2.trim.contigs.good.unique.good.filter.unique.precluster.pick.fasta)

mothur > classify.seqs(fasta=yanomami16s2.trim.contigs.good.unique.good.filter.unique.precluster.pick.fasta, count=yanomami16s2.trim.contigs.good.unique.good.filter.unique.precluster.denovo.vsearch.pick.count_table, reference=trainset14_032015.pds/trainset14_032015.pds.fasta, taxonomy=trainset14_032015.pds/trainset14_032015.pds.tax, cutoff=80)

mothur > remove.lineage(fasta=yanomami16s2.trim.contigs.good.unique.good.filter.unique.precluster.pick.fasta, count=yanomami16s2.trim.contigs.good.unique.good.filter.unique.precluster.denovo.vsearch.pick.count_table, taxonomy=yanomami16s2.trim.contigs.good.unique.good.filter.unique.precluster.pick.pds.wang.taxonomy, taxon=Chloroplast-Mitochondria-unknown-Eukaryota-Archea)

mothur > summary.tax()

mothur > dist.seqs(fasta=yanomami16s2.trim.contigs.good.unique.good.filter.unique.precluster.pick.pick.fasta, cutoff=0.20)

mothur > cluster(column=yanomami16s2.trim.contigs.good.unique.good.filter.unique.precluster.pick.pick.dist, count=yanomami16s2.trim.contigs.good.unique.good.filter.unique.precluster.denovo.vsearch.pick.pick.count_table)

mothur > make.shared(list=yanomami16s2.trim.contigs.good.unique.good.filter.unique.precluster.pick.pick.an.unique_list.list, count=yanomami16s2.trim.contigs.good.unique.good.filter.unique.precluster.denovo.vsearch.pick.pick.count_table, label=0.03)

mothur > classify.otu(list=yanomami16s2.trim.contigs.good.unique.good.filter.unique.precluster.pick.pick.an.unique_list.list, count=yanomami16s2.trim.contigs.good.unique.good.filter.unique.precluster.denovo.vsearch.pick.pick.count_table, taxonomy=yanomami16s2.trim.contigs.good.unique.good.filter.unique.precluster.pick.pds.wang.pick.taxonomy, label=0.03)

mothur > phylotype(taxonomy=yanomami16s2.trim.contigs.good.unique.good.filter.unique.precluster.pick.pick.an.unique_list.unique.cons.taxonomy)

mothur > make.shared(list=yanomami16s2.trim.contigs.good.unique.good.filter.unique.precluster.pick.pick.an.unique_list.0.09.pick.list, count=yanomami16s2.trim.contigs.good.unique.good.filter.unique.precluster.denovo.vsearch.pick.pick.pick.count_table, label=1)

mothur > classify.otu(list=yanomami16s2.trim.contigs.good.unique.good.filter.unique.precluster.pick.pick.an.unique_list.0.09.pick.list, count=yanomami16s2.trim.contigs.good.unique.good.filter.unique.precluster.denovo.vsearch.pick.pick.pick.count_table, taxonomy=yanomami16s2.trim.contigs.good.unique.good.filter.unique.precluster.pick.pds.wang.pick.taxonomy, label=1)

mothur > dist.seqs(fasta=yanomami16s2.trim.contigs.good.unique.good.filter.unique.precluster.pick.pick.fasta, output=lt, processors=60)

mothur > clearcut(phylip=yanomami16s2.trim.contigs.good.unique.good.filter.unique.precluster.pick.pick.phylip.dist)

mothur > system(mv yanomami16s2.trim.contigs.good.unique.good.filter.unique.precluster.pick.pick.phylip.tre yanomami16s2.tree)

mothur > system(mv yanomami16s2.trim.contigs.good.unique.good.filter.unique.precluster.denovo.vsearch.pick.pick.count_table yanomami16s2.count_table)

mothur > system(mv yanomami16s2.trim.contigs.good.unique.good.filter.unique.precluster.pick.pick.an.unique_list.shared yanomami16s2.shared)

mothur > system(mv yanomami16s2.trim.contigs.good.unique.good.filter.unique.precluster.pick.pick.an.unique_list.unique.cons.taxonomy yanomami16s2.constaxonomy)

mothur > count.groups(shared=yanomami16s2.trim.contigs.good.unique.good.filter.unique.precluster.pick.pick.an.unique_list.shared)

mothur > sub.sample(shared=yanomami16s2.shared, size=14732)

mothur > rarefaction.single(shared=yanomami16s2.trim.contigs.good.unique.good.filter.unique.precluster.pick.pick.an.unique_list.shared, calc=sobs, freq=100)

mothur > summary.single(shared=yanomami16s2.trim.contigs.good.unique.good.filter.unique.precluster.pick.pick.an.unique_list.shared, calc=nseqs-coverage-sobs-invsimpson-shannon-simpsoneven-chao-ace)

mothur > heatmap.bin(shared=yanomami16s2.0.03.subsample.shared, scale=log2, numotu=50)

mothur > dist.shared(shared=yanomami16s2.shared, calc=thetayc-jclass)

mothur > heatmap.sim(phylip=yanomami16s2.thetayc.unique.lt.dist)

mothur > heatmap.sim(phylip=yanomami16s2.jclass.unique.lt.dist)

mothur > venn(shared=yanomami16s2.shared, groups=G1-G2-G3-G4-G5-G6-G7-G8)

mothur > tree.shared(phylip=yanomami16s2.thetayc.unique.lt.dist)

mothur > parsimony(tree=yanomami16s2.thetayc.unique.lt.tre, group=yanomami16s2.design, groups=all)

mothur > pcoa(phylip=yanomami16s2.thetayc.unique.lt.dist)

mothur > nmds(phylip=yanomami16s2.thetayc.unique.lt.dist)

mothur > nmds(phylip=yanomami16s2.thetayc.unique.lt.dist, mindim=3, maxdim=3)

mothur > amova(phylip=yanomami16s2.thetayc.unique.lt.dist, design=yanomami16s2.design)

mothur > homova(phylip=yanomami16s2.thetayc.unique.lt.dist, design=yanomami16s2.design, processors=20)

mothur > get.communitytype(shared=yanomami16s2.shared)

mothur > metastats(shared=yanomami16s2.shared, design=yanomami16s2.design)

mothur > lefse(shared=yanomami16s2.shared, design=yanomami16s2.design)

mothur > indicator(shared=yanomami16s2.shared, design=yanomami16s2.design)

mothur > classify.rf(shared=yanomami16s2.shared, design=yanomami16s2.design)
