# Supplementary material for: Preliminary profile of the gut microbiota from amerindians in the Brazilian amazon experiencing a process of transition to urbanization
Source: Braz J Microbiol. 2024 Jun 24;55(3):2345–54. doi: 10.1007/s42770-024-01413-y (PMC11405645; doi:10.1007/s42770-024-01413-y)
Supplement: Supplementary file 2 — Supplementary Material 2 [file 42770_2024_1413_MOESM2_ESM.docx]

**Preliminary Profile of the Gut Microbiota from Amerindians in The Brazilian Amazon Experiencing a Process of Transition to Urbanization**

Brazilian Journal of Microbiology

Rodrigo M. Alencar^1^, José G. Martínez^1,2*^, Valéria N. Machado^1^, Juan F. Alzate^3^, Cinthya P. Ortiz-Ojeda^1,4^, Rosiane R. Matias^1^, Denise C. Benzaquem^1^, Maria C.F. Santos^1^, Enedina N. Assunção^5^, Evelyn C. Lira^5^, Spartaco Astolfi-Filho^5^, Tomas Hrbek^6^, Izeni P. Farias^6^, Cleiton Fantin^1^

^1^ Programa de Pós-graduação em Biotecnologia e Recursos Naturais da Amazônia, Universidade do Estado do Amazonas, Manaus, Brazil; ^2^ Grupo de investigación Biociencias, Facultad de Ciencias de la Salud, Institución Universitaria Colegio Mayor de Antioquia, Medellín, Colombia; ^3^ National Center for Genomic Sequencing, School of Medicine, Universidad de Antioquia, Medellín, Colombia; ^4^ Universidad Tecnológica del Perú, Lima, Peru; ^5^ Centro de Apoio Multidisciplinar, Universidade Federal do Amazonas, Manaus, Brazil; ^6^ Laboratório de Evolução e Genética Animal, Universidade Federal do Amazonas, Manaus, Brazil.

***** Corresponding author e-mail: [jose.martinez@colmayor.edu.co](mailto:jose.martinez@colmayor.edu.co)

**Online Resource 2.** Supplementary table reporting the demographic characteristics and Genbank accession codes of the study samples [Yanomami (Y) and Manaus (M) populations].

| Individual ID | Group | Age | Gender | Base | Next generation sequencing* |
| --- | --- | --- | --- | --- | --- |
| Y 1 | G1 | 4 m | Female | Marari | SAMN26417896 |
| Y 3 | G3 | 20 y | Male | Marari | SAMN26417898 |
| Y 4 | G4 | 65 y | Female | Marari | SAMN26417899 |
| Y 5 | G3 | 32 y | Male | Toototobi | SAMN26417898 |
| Y 6 | G4 | 52 y | Male | Toototobi | SAMN26417899 |
| Y 7 | G2 | 1 y | Male | Xitei | SAMN26417897 |
| Y 8 | G2 | 1 y | Male | Xitei | SAMN26417897 |
| Y 9 | - | 2 m | Male | Surucucu | - |
| Y 10 | G2 | 2 y | Male | Surucucu | SAMN26417897 |
| Y 12 | G1 | 9 m | Female | Aratha-Ú | SAMN26417896 |
| Y 13 | G2 | 7 y | Male | Aratha-Ú | SAMN26417897 |
| Y 14 | G2 | 5 y | Female | Novo Demini | SAMN26417897 |
| Y 15 | G3 | 16 y | Female | Novo Demini | SAMN26417898 |
| Y 16 | G3 | 34 y | Female | Novo Demini | SAMN26417898 |
| Y 17 | G4 | 50 y | Female | Kayana-Ú | SAMN26417899 |
| Y 18 | G4 | 62 y | Male | Kayana-Ú | SAMN26417899 |
| Y 19 | G2 | 2 y | Male | Maloca Paapi-Ú | SAMN26417897 |
| Y 20 | G4 | 55 y | Male | Waputha | SAMN26417899 |
| Y 21 | G3 | 30 y | Male | Maloca Paapi-Ú | SAMN26417898 |
| M 22 | G6 | 7 y | Male | Manaus | SAMN26417901 |
| M 24 | G7 | 14 y | Female | Manaus | SAMN26417902 |
| M 25 | G7 | 34 y | Male | Manaus | SAMN26417902 |
| M 26 | G8 | 47 y | Female | Manaus | SAMN26417903 |
| M 27 | - | 50 y | Male | Manaus | - |
| M 28 | G7 | 17 y | Female | Manaus | SAMN26417902 |
| M 29 | G6 | 1 y | Male | Manaus | SAMN26417901 |
| M 30 | G6 | 5 y | Male | Manaus | SAMN26417901 |
| M 31 | G8 | 50 y | Female | Manaus | SAMN26417903 |
| M 32 | G6 | 7 y | Female | Manaus | SAMN26417901 |
| M 33 | G5 | 5 m | Male | Manaus | SAMN26417900 |
| M 38 | G7 | 37 y | Female | Manaus | SAMN26417902 |
| M 41 | G8 | 62 y | Female | Manaus | SAMN26417903 |

Age was expressed in years (y) and months (m).

* Sequence Read Archive (SRA) BioProject PRJNA812515 (16S rRNA metagenomic approach).
