# Supplementary material for: Preliminary profile of the gut microbiota from amerindians in the Brazilian amazon experiencing a process of transition to urbanization
Source: Braz J Microbiol. 2024 Jun 24;55(3):2345–54. doi: 10.1007/s42770-024-01413-y (PMC11405645; doi:10.1007/s42770-024-01413-y)
Supplement: Supplementary file 3 — Supplementary Material 3 [file 42770_2024_1413_MOESM3_ESM.docx]

**Preliminary Profile of the Gut Microbiota from Amerindians in The Brazilian Amazon Experiencing a Process of Transition to Urbanization**

Brazilian Journal of Microbiology

Rodrigo M. Alencar^1^, José G. Martínez^1,2*^, Valéria N. Machado^1^, Juan F. Alzate^3^, Cinthya P. Ortiz-Ojeda^1,4^, Rosiane R. Matias^1^, Denise C. Benzaquem^1^, Maria C.F. Santos^1^, Enedina N. Assunção^5^, Evelyn C. Lira^5^, Spartaco Astolfi-Filho^5^, Tomas Hrbek^6^, Izeni P. Farias^6^, Cleiton Fantin^1^

^1^ Programa de Pós-graduação em Biotecnologia e Recursos Naturais da Amazônia, Universidade do Estado do Amazonas, Manaus, Brazil; ^2^ Grupo de investigación Biociencias, Facultad de Ciencias de la Salud, Institución Universitaria Colegio Mayor de Antioquia, Medellín, Colombia; ^3^ National Center for Genomic Sequencing, School of Medicine, Universidad de Antioquia, Medellín, Colombia; ^4^ Universidad Tecnológica del Perú, Lima, Peru; ^5^ Centro de Apoio Multidisciplinar, Universidade Federal do Amazonas, Manaus, Brazil; ^6^ Laboratório de Evolução e Genética Animal, Universidade Federal do Amazonas, Manaus, Brazil.

***** Corresponding author e-mail: [jose.martinez@colmayor.edu.co](mailto:jose.martinez@colmayor.edu.co)

**Online Resource 3.** Supplementary table reporting the relative abundance of genera (OTUs) found across study groups [Yanomami (G1-G4) and Manaus (G5-G8) populations] using the Mothur software.

| **Genera** | **G1** | **G2** | **G3** | **G4** | **G5** | **G6** | **G7** | **G8** |
| --- | --- | --- | --- | --- | --- | --- | --- | --- |
| Corynebacterium | 0 | 0.000273261 | 0 | 0 | 0 | 0.000122026 | 0 | 0 |
| Micrococcus | 0 | 0 | 0 | 0.000154536 | 0 | 0.000122026 | 0.000306796 | 0 |
| Propionibacterium | 0.000345304 | 0.000546523 | 0.000423191 | 0.000618142 | 0 | 0.000488103 | 0.000613591 | 0.000429984 |
| Bifidobacterium | 0.000345304 | 0.000683153 | 0.001128509 | 0.001236285 | 0 | 0.000610128 | 0.000766989 | 0.000429984 |
| Olsenella | 0 | 0.000136631 | 0.000141064 | 0 | 0.000474158 | 0.000122026 | 0 | 0 |
| Bacteroides | 0.003798343 | 0.003142506 | 0.009874453 | 0.00633596 | 0.341868184 | 0.061622941 | 0.120263844 | 0.14762792 |
| Barnesiella | 0.000345304 | 0 | 0 | 0 | 0 | 0 | 0.007669888 | 0 |
| Butyricimonas | 0 | 0.000136631 | 0.000282127 | 0.000309071 | 0 | 0.001830384 | 0.00276116 | 0.002436577 |
| Odoribacter | 0 | 0.000409892 | 0.000564254 | 0.000463607 | 0 | 0.002684564 | 0.004295137 | 0.001576609 |
| Parabacteroides | 0.002762431 | 0.003415767 | 0.009169135 | 0.006026889 | 0.009009009 | 0.036363636 | 0.01303881 | 0.039271893 |
| Alloprevotella | 0.000690608 | 0.000409892 | 0.000282127 | 0.000309071 | 0 | 0.000244051 | 0.001227182 | 0.001146625 |
| Paraprevotella | 0 | 0.000546523 | 0.000282127 | 0.000618142 | 0 | 0.000122026 | 0.000613591 | 0.001719937 |
| Prevotella | 0.892265193 | 0.772509906 | 0.743546339 | 0.709782105 | 0.52916074 | 0.742403905 | 0.514802884 | 0.415651426 |
| Alistipes | 0.000345304 | 0.000136631 | 0.000282127 | 0.000309071 | 0.000474158 | 0.012812691 | 0.035741678 | 0.000859968 |
| Enterococcus | 0.000690608 | 0.000136631 | 0.000282127 | 0.000309071 | 0 | 0.000244051 | 0.000153398 | 0.000143328 |
| Lactobacillus | 0.005524862 | 0.058887826 | 0.034842714 | 0.068613815 | 0.00284495 | 0.004148871 | 0.027304801 | 0.006879748 |
| Streptococcus | 0.000690608 | 0.000546523 | 0.000282127 | 0.000463607 | 0.000948317 | 0.000244051 | 0.000460193 | 0.000429984 |
| Clostridium_sensu_stricto | 0.000345304 | 0.002186091 | 0.002257018 | 0.003090712 | 0 | 0.001708359 | 0.003374751 | 0.001146625 |
| Anaerovorax | 0 | 0.000136631 | 0.000423191 | 0.001545356 | 0 | 0.000366077 | 0.001994171 | 0.000286656 |
| Anaerostipes | 0 | 0 | 0 | 0 | 0.000474158 | 0 | 0 | 0 |
| Blautia | 0.002762431 | 0.025959831 | 0.048666949 | 0.032607016 | 0.016121385 | 0.013788896 | 0.024697039 | 0.024079117 |
| Butyrivibrio | 0 | 0.000273261 | 0.000282127 | 0.000309071 | 0 | 0 | 0 | 0.000286656 |
| Clostridium_XlVa | 0 | 0 | 0 | 0 | 0.000474158 | 0.000244051 | 0.000613591 | 0.002293249 |
| Clostridium_XlVb | 0 | 0.000956415 | 0.002115954 | 0.002627106 | 0 | 0.001098231 | 0.002454364 | 0.004156514 |
| Coprococcus | 0.000690608 | 0.001229676 | 0.001410636 | 0.002627106 | 0 | 0.001098231 | 0.007976684 | 0.001289953 |
| Dorea | 0.001035912 | 0.00997404 | 0.007758499 | 0.012980992 | 0 | 0.006345333 | 0.011504832 | 0.005876451 |
| Oribacterium | 0 | 0 | 0 | 0.000154536 | 0 | 0.000122026 | 0 | 0 |
| Roseburia | 0.001381215 | 0.010383932 | 0.038792495 | 0.013444599 | 0 | 0.005247102 | 0.014419389 | 0.116955712 |
| Ruminococcus2 | 0.000690608 | 0.003005875 | 0.005501481 | 0.004790604 | 0.000948317 | 0.003416718 | 0.016106765 | 0.014332808 |
| Clostridium_XI | 0 | 0.000409892 | 0.0015517 | 0.000772678 | 0 | 0.000610128 | 0.003221353 | 0.000573312 |
| Peptostreptococcus | 0 | 0.000273261 | 0.000282127 | 0 | 0 | 0 | 0 | 0 |
| Anaerotruncus | 0 | 0 | 0.000141064 | 0 | 0 | 0.000122026 | 0.000460193 | 0.001003297 |
| Butyricicoccus | 0.000690608 | 0.000409892 | 0.000282127 | 0.000618142 | 0 | 0.000366077 | 0.000766989 | 0.000573312 |
| Faecalibacterium | 0.015538674 | 0.03374778 | 0.032867823 | 0.04481533 | 0.000474158 | 0.035753508 | 0.044792146 | 0.11709904 |
| Flavonifractor | 0 | 0 | 0 | 0 | 0.000948317 | 0 | 0 | 0.000429984 |
| Oscillibacter | 0.011740331 | 0.005055335 | 0.005219354 | 0.007572245 | 0.005215742 | 0.006833435 | 0.024390244 | 0.012756199 |
| Pseudoflavonifractor | 0 | 0 | 0 | 0 | 0 | 0 | 0.000306796 | 0 |
| Ruminococcus | 0 | 0.001093045 | 0.001128509 | 0.005563282 | 0 | 0.002928615 | 0.057063967 | 0.02164254 |
| Subdoligranulum | 0.000345304 | 0.000136631 | 0.000141064 | 0.000927214 | 0 | 0.000488103 | 0.005215524 | 0.004299842 |
| Catenibacterium | 0 | 0 | 0 | 0 | 0 | 0 | 0.000153398 | 0.000143328 |
| Clostridium_XVIII | 0 | 0 | 0 | 0 | 0.004267425 | 0 | 0 | 0 |
| Dialister | 0 | 0 | 0 | 0.000154536 | 0.002370792 | 0 | 0.000153398 | 0 |
| Megamonas | 0 | 0 | 0 | 0 | 0.014224751 | 0 | 0 | 0 |
| Megasphaera | 0.000345304 | 0.000136631 | 0.000141064 | 0.000309071 | 0.032716927 | 0.000122026 | 0.000153398 | 0.020352587 |
| Mitsuokella | 0 | 0 | 0 | 0 | 0 | 0 | 0 | 0.001576609 |
| Veillonella | 0.010359116 | 0.00792458 | 0.004655099 | 0.009426673 | 0.019914651 | 0.004636974 | 0.003221353 | 0.002006593 |
| Victivallis | 0 | 0 | 0 | 0 | 0.000474158 | 0 | 0 | 0 |
| Parasutterella | 0 | 0 | 0 | 0 | 0 | 0 | 0.000613591 | 0 |
| Sutterella | 0.018646409 | 0.015712529 | 0.01128509 | 0.017462525 | 0.006638217 | 0.014399024 | 0.007823286 | 0.009889637 |
| Campylobacter | 0.027279006 | 0.030878535 | 0.025673579 | 0.031525267 | 0.009009009 | 0.029530201 | 0.029145574 | 0.014762792 |
| Succinivibrio | 0 | 0 | 0.000987445 | 0 | 0 | 0 | 0 | 0 |
| Escherichia/Shigella | 0.000345304 | 0.008197841 | 0.007053181 | 0.010199351 | 0.000948317 | 0.006589384 | 0.009203866 | 0.003583202 |
| Treponema | 0 | 0 | 0 | 0.000927214 | 0 | 0 | 0 | 0 |
| Cloacibacillus | 0 | 0 | 0 | 0 | 0 | 0 | 0.000153398 | 0 |

**Online Resource 4**. Supplementary figure showing the comparative analyses of Firmicutes/Bacteroidetes ratio found across the study groups [Yanomami and Manaus], in other non-industrialized and industrialized populations, reviewed by Magne et al. (2020) and originally obtained by Kann et al. (2023). Ratios are expressed as medians.
