# Supplementary figures and images for: Preliminary profile of the gut microbiota from amerindians in the Brazilian amazon experiencing a process of transition to urbanization
Source: Braz J Microbiol. 2024 Jun 24;55(3):2345–54. doi: 10.1007/s42770-024-01413-y (PMC11405645; doi:10.1007/s42770-024-01413-y)

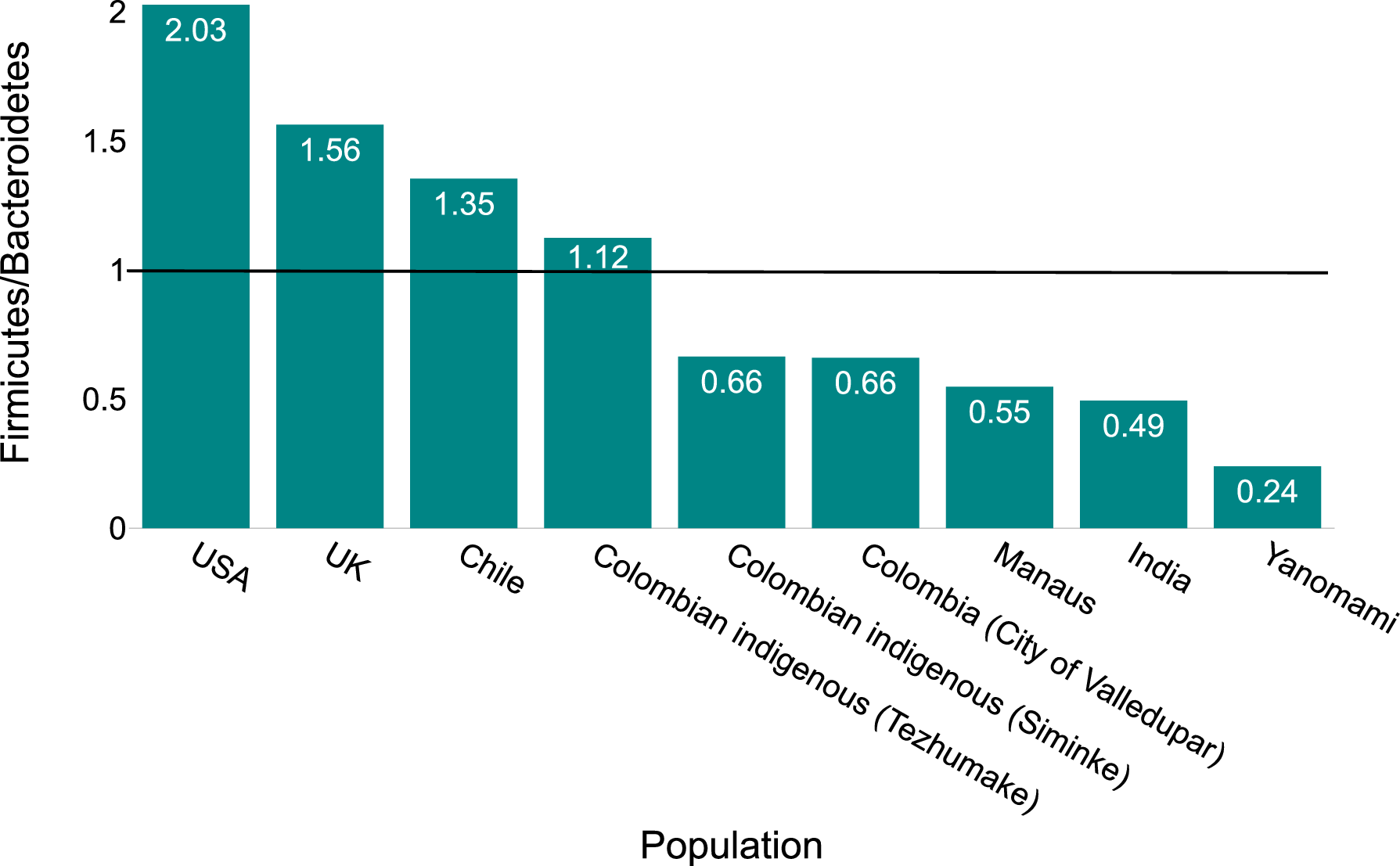

Supplement: Supplementary file 4 — Supplementary Material 4 [file 42770_2024_1413_MOESM4_ESM.tiff]
